# *Fusobacterium nucleatum* Subspecies Differ in Biofilm Forming Ability *in vitro*

**DOI:** 10.3389/froh.2022.853618

**Published:** 2022-03-15

**Authors:** Maria Muchova, Dario L. Balacco, Melissa M. Grant, Iain L. C. Chapple, Sarah A. Kuehne, Josefine Hirschfeld

**Affiliations:** Periodontal Research Group, School of Dentistry, Institute of Clinical Sciences, University of Birmingham, Birmingham, United Kingdom

**Keywords:** biofilm, *Fusobacterium nucleatum*, periodontitis, subspecies, pathogen, fusobacteria, adhesin

## Abstract

Development of dysbiosis in complex multispecies bacterial biofilms forming on teeth, known as dental plaque, is one of the factors causing periodontitis. *Fusobacterium nucleatum* (*F. nucleatum*) is recognised as a key microorganism in subgingival dental plaque, and is linked to periodontitis as well as colorectal cancer and systemic diseases. Five subspecies of *F. nucleatum* have been identified: *animalis, fusiforme, nucleatum, polymorphum*, and *vincentii*. Differential integration of subspecies into multispecies biofilm models has been reported, however, biofilm forming ability of individual *F. nucleatum* subspecies is largely unknown. The aim of this study was to determine the single-subspecies biofilm forming abilities of *F. nucleatum* ATCC type strains. Static single subspecies *F. nucleatum* biofilms were grown anaerobically for 3 days on untreated or surface-modified (sandblasting, artificial saliva, fibronectin, gelatin, or poly-L-lysine coating) plastic and glass coverslips. Biofilm mass was quantified using crystal violet (CV) staining. Biofilm architecture and thickness were analysed by scanning electron microscopy and confocal laser scanning microscopy. Bioinformatic analysis was performed to identify orthologues of known adhesion proteins in *F. nucleatum* subspecies. Surface type and treatment significantly influenced single-subspecies biofilm formation. Biofilm formation was overall highest on poly-L-lysine coated surfaces and sandblasted glass surfaces. Biofilm thickness and stability, as well as architecture, varied amongst the subspecies. Interestingly, *F. nucleatum* ssp. *polymorphum* did not form a detectable, continuous layer of biofilm on any of the tested substrates. Consistent with limited biofilm forming ability *in vitro, F. nucleatum* ssp. *polymorphum* showed the least conservation of the adhesion proteins CmpA and Fap2 *in silico*. Here, we show that biofilm formation by *F. nucleatum in vitro* is subspecies- and substrate-specific. Additionally, *F. nucleatum* ssp. *polymorphum* does not appear to form stable single-subspecies continuous layers of biofilm *in vitro*. Understanding the differences in *F. nucleatum* single-subspecies biofilm formation may shed light on multi-species biofilm formation mechanisms and may reveal new virulence factors as novel therapeutic targets for prevention and treatment of *F. nucleatum*-mediated infections and diseases.

## Introduction

The oral environment consists of a multitude of bacterial species living on both soft and hard tissues in complex multispecies communities known as biofilms [1]. Dental plaque, a type of biofilm which forms on the surface of teeth, has been extensively studied for decades due to its association with periodontitis, a chronic inflammatory disease of the tooth-supporting tissues [2]. Periodontitis causes connective tissue attachment loss, inflammation and, if left untreated, tooth loss [2, 3]. One of the factors leading to periodontitis is the accumulation of dental plaque and emergence of dysbiosis therein [2, 3].

Health-related supra- and sub-gingival bacterial communities are often composed of Gramme-positive and Gram-negative early colonisers including *Streptococcus, Neisseria, Prevotella, Haemophilus*, and *Rothia* genera, among other less abundant genera, creating a distinct signature of a healthy, symbiotic state [4–7]. However, studies analysing diseased periodontal sites exhibited a shift toward communities containing anaerobic, Gram-negative periodontal pathogens belonging to Socransky's “red complex” and leading to dysbiosis with an associated destructive immune-inflammatory response resulting in tissue damage [8–12].

*Fusobacterium nucleatum*, an anaerobic commensal member of dental biofilms [13], is present in low numbers in healthy subgingival dental biofilm [4] but is enriched in periodontal pockets [14]. It has been identified as a key bridging organism between the early colonisers and periodontal pathogens [15]. It is also considered an opportunistic pathogen due to its association with systemic diseases, such as cardiovascular disease, ulcerative colitis and colorectal cancer [13, 16], as well as with extra-oral infections which can lead to adverse pregnancy outcomes [13]. Five subspecies of *F. nucleatum* have been identified to date: *animalis, fusiforme, nucleatum, polymorphum*, and *vincentii* [13]. Some authors only recognise four subspecies, classifying *fusiforme* and *vincentii* as one subspecies *vincentii* [17].

Despite the close genomic relatedness of all subspecies, differences in pathogenicity have been recognised, such as host immune response modulation: subspecies *nucleatum* (ATCC 25586), *polymorphum* (ATCC 10953) and *vincentii* (ATCC 49256) were shown to prevent superoxide generation induced by N-formylmethionyl-leucyl-phenylalanine in neutrophil-like HL-60 cells. Additionally, subspecies *polymorphum* increased the rate of apoptosis in HL-60 cells when compared to the other subspecies [18]. *F. nucleatum* subspecies were also studied in *in vitro* multi-species biofilm models. Differences in the incorporation of subspecies into the biofilm were described, where bacterial numbers of *F. nucleatum* ssp. *vincentii* and ssp. *polymorphum* were significantly lower in comparison with ssp. *nucleatum* [19].

*F. nucleatum* adhesion proteins have been identified, which mediate coaggregation and biofilm formation with various oral microbes as well as salivary proteins: outer membrane protein RadD with a putative accessory protein Aid1 [20], autotransporter Fap2 [20], porin FomA [20], outer membrane protein CmpA [20], and adhesin FadA [21]. Homologues of adhesins from other bacterial species have also been identified in *F. nucleatum in silico*, such as YadA-like adhesin originally found in *Yersinia* species [22].

Surprisingly, despite its structural importance in dental plaque and its role in pathogenic biofilms in colorectal cancer and other diseases, biofilm formation by single *F. nucleatum* subspecies is poorly understood. Moreover, few authors have used type strains as reference strains to the clinical isolates in studies of *F. nucleatum*, and many have not reported the subspecies used, making a comparison of these results difficult. While it is clinically relevant to study *F. nucleatum* in multi-species biofilms, information obtained from single-subspecies *F. nucleatum* biofilms without the presence of additional binding partners is necessary for better understanding of biofilm-related immunogenic and pathogenic properties of *F. nucleatum* subspecies and virulence factor expression.

Therefore, this study aimed to investigate single-subspecies biofilm formation abilities by *F. nucleatum* subspecies along with analysis of differences in adhesins known to mediate biofilm formation and aggregation. A further aim was to utilise a simplified *in vitro* single-subspecies biofilm model which could be repeated by other researchers, using widely available substrates and substrate coatings, to better understand biological properties of each subspecies. We hypothesised that there is no difference in biofilm formation and architecture among *F. nucleatum* subspecies.

## Materials and Methods

### Bacterial Strains and Growth Conditions

The following type strains of *F. nucleatum* were obtained from the Periodontal Research Group culture collection (School of Dentistry, University of Birmingham, UK) and were originally purchased from the American Tissue Culture Collection (ATCC): *F. nucleatum* ssp. *animalis* ATCC 51191 (FNA), *F. nucleatum* ssp. *fusiforme* ATCC 51190 (FNF), *F. nucleatum* ssp. *nucleatum* ATCC 25586 (FNN25), *F. nucleatum* ssp. *polymorphum* ATCC 10593 (FNP), and *F. nucleatum* ssp. *vincentii* ATCC 49256 (FNV). Additionally, a genetically tractable strain *F. nucleatum* ssp. *nucleatum* ATCC 23726 (FNN23) was kindly provided by Dr. Daniel Slade (Virginia Polytechnic Institute and State University, Blacksburg VA, USA). The identity of all subspecies was confirmed by 16S rRNA sequencing.

All subspecies were grown at 37°C in an anaerobic chamber (80% N_2_, 10% CO_2_, and 10% H_2_; Don Whitley DG250 Anaerobic Workstation, Don Whitley Scientific, Bingley, UK) on Schaedler anaerobe agar plates (SAA; Sigma-Aldrich/Merck, Darmstadt, Germany). Liquid cultures were grown in Schaedler anaerobe broth (SAB; Oxoid, Basingstoke, UK).

### Surface Coating and Biofilm Growth

In order to evaluate biofilm formation of *F. nucleatum* subspecies, substrates commonly used for *in vitro* biofilm studies were used: glass (12 mm diameter; Marienfeld Superior, Lauda-Königshofen, Germany) and plastic (13 mm diameter; Thermo Scientific™ Nunc™ Thermanox™, Thermo Fisher Scientific, Loughborough, UK; referred to as “Thermanox” hereafter) coverslips placed in a 24-well-plate. Artificial saliva (AS) was used as a coating agent to mimic human saliva and formation of salivary pellicle to promote bacterial adhesion [23]. AS was prepared according to Millhouse et al. [24] by using the following reagents: porcine stomach mucins 0.25% w/v, potassium chloride 0.02% w/v, calcium chloride dihydrate 0.02% w/v, yeast extract 0.2% w/v, proteose peptone 0.5% w/v (all obtained from Sigma-Aldrich/Merck, Darmstadt, Germany), sodium chloride 0.35% w/v (Thermo Fisher Scientific, Loughborough, UK), and Lab-Lemco powder 0.1% w/v (Oxoid, Basingstoke, UK) in ultrapure water (Milli-Q, Merck Millipore, Burlington, MA, USA); urea was added after autoclaving to a final concentration of 0.05% v/v (Sigma-Aldrich). Additionally, substrates were coated using agents promoting cell attachment to culture surfaces: fibronectin (from human plasma, Merck Millipore) [25], gelatin (from porcine skin) [26], and poly-L-lysine (both from Sigma-Aldrich/Merck) [27].

Substrate coating was performed as follows: AS was added to coverslips and incubated at 37°C for 1 h [28]. Next, AS was removed and bacterial cultures were added immediately without substrate washing or drying to simulate *in vivo* conditions. Fibronectin diluted in PBS (5 μg/cm^2^) was applied and incubated for 45 min at room temperature. Gelatin solution (0.1% in PBS) was incubated for 20 min at 37°C. Poly-L-lysine coating was performed for 10 min at room temperature. These surface coatings were left to dry in a laminar flow hood as per the manufacturers' instructions without washing. Additionally, a set of glass coverslips was sandblasted to create a roughened surface. Sandblasting was completed using a Basic quattro sandblasting unit (Renfert, Hilzingen, Germany), in which each coverslip was treated for 5 s with aluminium oxide (25 μm grit size, 1.2 mm nozzle).

To initiate biofilm growth, planktonic *F. nucleatum* cultures were washed once with PBS and the optical density of each culture was adjusted to OD_600_ = 1 in SAB, corresponding to 1.62 × 10^9^ CFU/ml for each subspecies. For each single-subspecies biofilm, 400 μl of bacterial suspension was added to each well containing studied substrates and biofilms were incubated static for 72 h, under anaerobic conditions. SAB was replaced after 24 and 48 h and biofilms were monitored for contamination daily.

### Biomass Quantification Using Crystal Violet

Biofilm biomass was quantified by crystal violet (CV) staining. After 72 h of incubation, biofilms were carefully washed once with 100 μl PBS and air-dried for 2 h at 37°C, then stained with a CV solution (200 μl, 0.05 % w/v) at room temperature for 30 min. After staining, biofilms were gently washed with 200 μl PBS and air dried at 37°C for 2 h. Ethanol (100%, 200 μl) was used to destain the biofilms for 1 h on a plate shaker. Ethanol solution from each well was diluted in Milli-Q water 1:10 in a 96-well plate and the absorbance was measured at 600 nm (Microplate reader Spark^®^, Tecan; software SparkControl, v. 2.3, Tecan). To account for differences between glass and plastic coverslip surface area, absorbance readings per cm^2^ were calculated using the formula below. The surface area of a glass coverslip was 113.04 mm^2^, the area of a Thermanox coverslip was 132.67 mm^2^.


$$\begin{array}{l} average\;CV\;absorbance\;corrected\;for\;blank\;\times \frac{{100\;mm}^{2}}{area\;of\;the\;coverslip\;\left(mm^{2}\right)} \end{array}$$

### Fluorescent Biofilm Staining and Confocal Laser Scanning Microscopy (CLSM)

Biofilms for CLSM analysis were grown in 24-well black plates with clear base (polystyrene, thickness 190 μm, Vision Plate™, 4titude, Surrey, UK) either with no surface treatment, or coated with fibronectin, gelatin or poly-L-lysine as stated above. In order to avoid excessive detachment, biofilms were first fixed with 4% paraformaldehyde for 10 min, then washed in PBS and stained using green fluorescent, cell permeant nucleic acid stain SYTO™ 9 (FilmTracer™ LIVE/DEAD^®^ Biofilm Viability Kit, Invitrogen, Renfrew, UK) according to the manufacturer's instructions. Briefly, 3 μl of SYTO™ 9 were added to 1 ml of sterile PBS and biofilms were stained with 200 μl of the diluted stain for 20 min at room temperature in the dark.

Samples were imaged immediately after staining using CLSM (LSM 700, Zeiss, Germany), with a 40X oil immersion objective at 488/500 nm. Maximum thickness of the biofilms was estimated by obtaining z-stack horizontal images at 1.3 μm intervals. Biofilms were grown in triplicate in one experiment and images were acquired in the centre of each well using Zeiss Zen 2011 software.

### Preparation of Biofilms and Scanning Electron Microscopy (SEM)

In order to examine single-subspecies biofilm architecture using SEM, biofilms were grown on poly-L-lysine coated plastic (Thermanox™) coverslips in 24-well plates. Biofilms were fixed using 2.5% glutaraldehyde (Agar Scientific, Stansted, United Kingdom) in 0.1 M sodium cacodylate buffer (pH 7.4, BioWorld, Dublin, Ireland) for 10 min at room temperature. Following fixation, biofilms were dehydrated with increasing ethanol concentrations (20–100%) and incubated for 10 min at each step.

Finally, drying agent hexamethyldisilizane (Sigma-Aldrich/Merck, Darmstadt, Germany) was applied and left to evaporate overnight. Coverslips with biofilms were mounted onto aluminium specimen stubs (Agar Scientific, Stansted, United Kingdom), sputter coated with two layers of gold and visualised using a scanning electron microscope (Zeiss EVO MA10). A visible layer of biofilm was chosen after visual evaluation of each specimen under low magnification (50X) and recorded at 1000X and 5000X magnification.

### Bioinformatics and Phylogenetic Analyses

Bacterial genomes of *F. nucleatum* ssp. *animalis* ATCC 51191 (GCA_000220825), *F. nucleatum* ssp. *nucleatum* ATCC 23726 (GCA_000178895), *F. nucleatum* ssp. *nucleatum* ATCC 25586 (GCA_000007325), *F. nucleatum* ssp. *polymorphum* ATCC 10953 (GCA_000153625), *F. nucleatum* ssp. *vincentii* ATCC 49256 (GCA_000182945) were retrieved from EnsemblBacteria (Release 52) [29]. *F. nucleatum* ssp. *fusiforme* data were not available on this portal, possibly due to their close genetic relatedness with *F. nucleatum* ssp. *vincentii*.

Orthologues were identified using blastp (v. 2.12.0) [30] with default parameters and *F. nucleatum*, ssp. *nucleatum* ATCC 25586 proteins as queries, with a cutoff e-value of 1e-10. Protein domains were annotated using InterPro [31] with a cutoff e-value of 1e-5. Multisequence alignment of CmpA and Fap2 proteins was performed using MAFFT (v. 6) [32] with the “– auto” option. The phylogenetic tree was constructed using the Neighbour-Joining method [33], JTT substitution model and bootstrap 1000.

### Statistical Analysis

Statistical analysis was performed using GraphPad Prism (version 9.3.0 for Windows, GraphPad Software, San Diego, California, USA). Biomass quantification data were analysed using Shapiro-Wilk normality test and shown to conform to normal distribution. Samples were compared within subspecies and either untreated Thermanox™ surface or untreated glass surface were considered as controls. Values were analysed by one-way ANOVA, followed by Dunnett's *post-hoc* test. Results were considered statistically significant if *p* < 0.05. Graphs were created using GraphPad Prism, version 9.3.0.

## Results

### Biofilm Thickness and Stability Varies Among *F. nucleatum* Subspecies and on Different Surfaces

All subspecies formed biofilms detectable with CV, with the exception of FNP, which did not form a continuous biofilm on any of the tested surfaces. When examined visually during the incubation period, this subspecies was found to remain planktonic in the biofilm supernatant.

**Figure 1:**
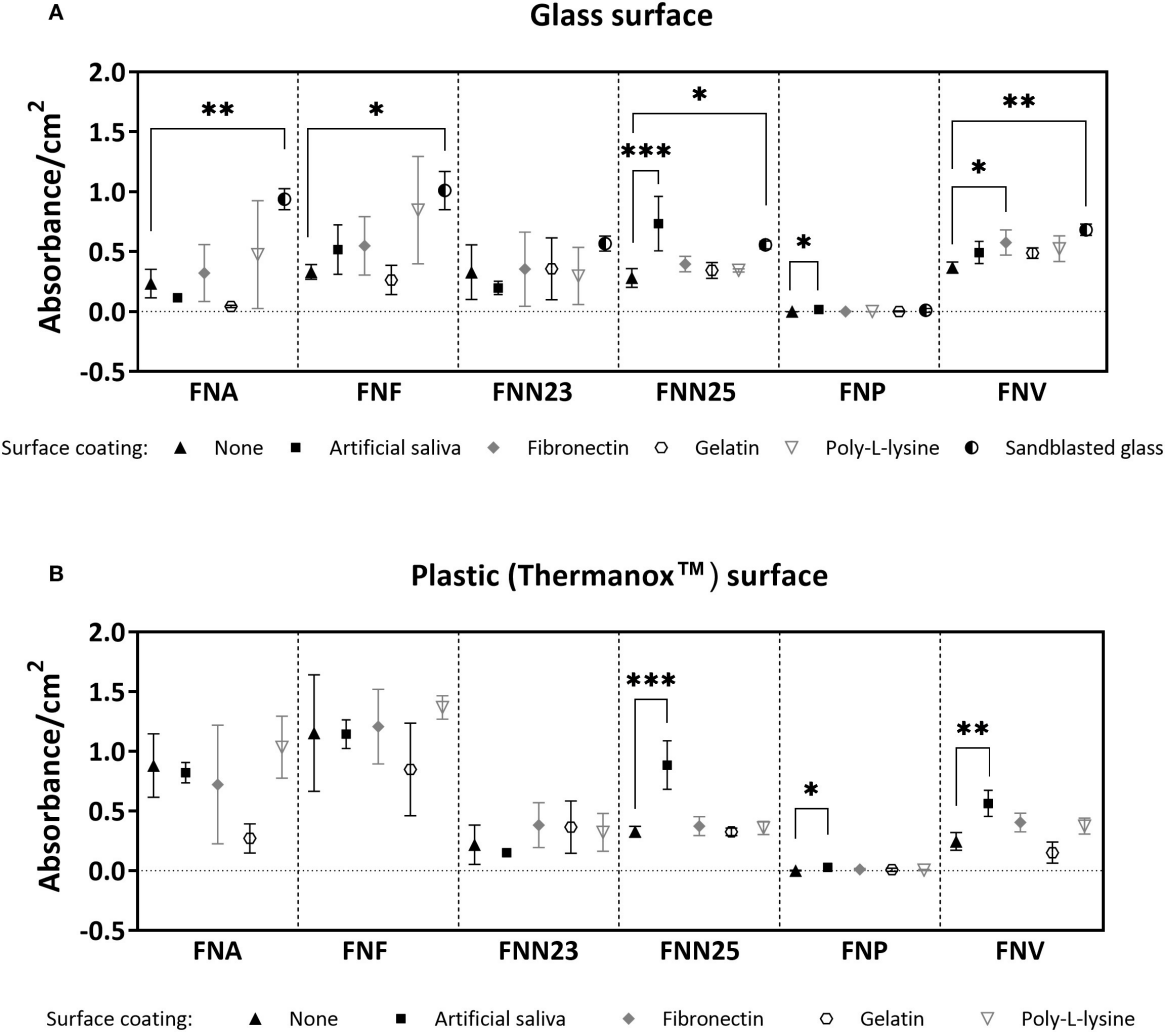
Crystal violet biomass quantification results. **(A)** Quantification on glass coverslips with or without (control) surface coatings. No significant differences were found for FNN23. **(B)** Quantification on Thermanox coverslips with or without (control) surface coatings. No significant differences were found for FNA, FNF, and FNN23. Assays were carried out as three independent experiments in triplicate. Mean values with standard deviations are shown. One-way ANOVA was performed followed by Dunnett's *post-hoc* test for within subspecies differences between control (uncoated glass/uncoated Thermanox) and test samples (*p < 0.05; **p < 0.01; ***p < 0.001).

Differences in biofilm mass were seen amongst the subspecies and on the different surfaces (Figures 1A,B): Generally, sandblasted glass surfaces supported biofilm formation best with significantly higher biofilm mass in most subspecies compared to untreated glass. Second best surface coating was AS, supporting significantly higher biofilm formation by FNN25 and FNP on glass surfaces, and FNN25, FNP, and FNV on plastic surfaces. However, even though absorbance values of FNP biofilms were significantly higher on both glass and plastic coated with AS compared to untreated surfaces (*p* = 0.04 and *p* = 0.02, respectively), the amount of biofilm quantified was consistently low (mean absorbance values of 0.017 and 0.029, respectively). FNF adhered significantly better to uncoated Thermanox coverslips when compared to uncoated glass (*p* = 0.002), however the other subspecies showed no difference in biofilm formation between glass and plastic surfaces. FNA and FNF were the best biofilm formers on plastic surfaces.

**Figure 2:**
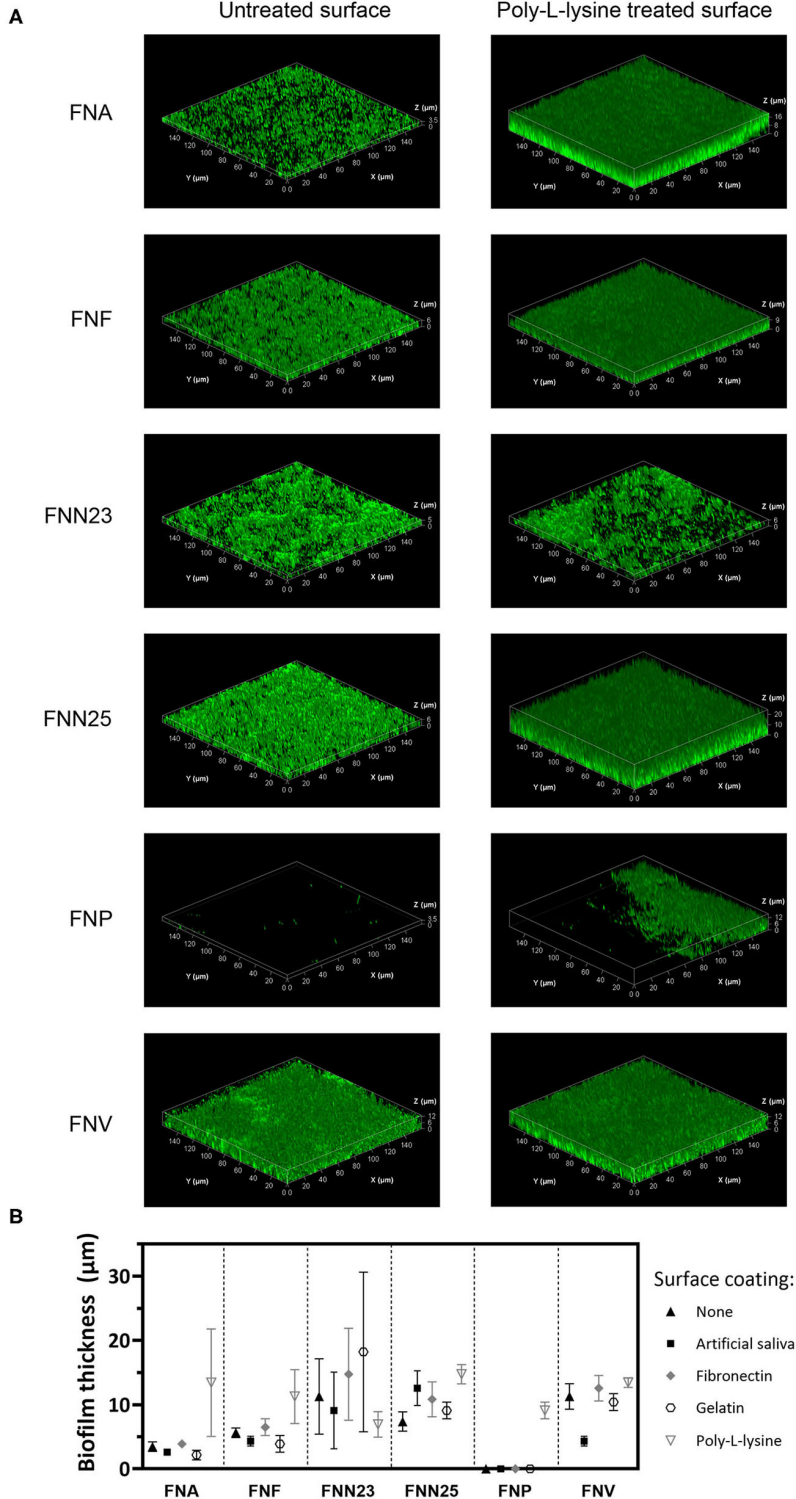
Biofilm thickness of *F. nucleatum* subspecies determined by CLSM. **(A)** Representative Z-stack 3D images of single-subspecies biofilms grown on poly-L-lysine coated plastic surface. Biofilms are enclosed in a bounding box with scaled coordinates; x, y, and z axes show the dimensions indicated in μm. Differences in biofilm thickness can be observed. **(B)** Biofilm thickness estimated from z-stacks. Experiment was performed once with biofilms grown in triplicates. Mean values with standard deviations are shown.

Biofilm thickness was also examined using CLSM and mean values estimated from three-dimensional biofilm images (Figure 2). Similar to the results obtained by CV staining, all subspecies except FNP formed a continuous layer of biofilm. Only single bacterial cells of FNP were observed on untreated and coated surfaces (Supplementary Figure 1). A small area of biofilm formation by FNP was detected in the centre of poly-L-lysine coated wells, for which the thickness was determined (Figure 2B; 9.1 ±1.3 μm, mean ± SD).

Large standard deviations were associated with some subspecies-surface combinations (FNA, FNF) and were seen in both CV and CLSM experiments. These reflect a high degree of visually observed biofilm detachment during handling, indicating low adhesive strength of these biofilms.

### Biofilm Architecture Differs Among *F. nucleatum* Subspecies

**Figure 3:**
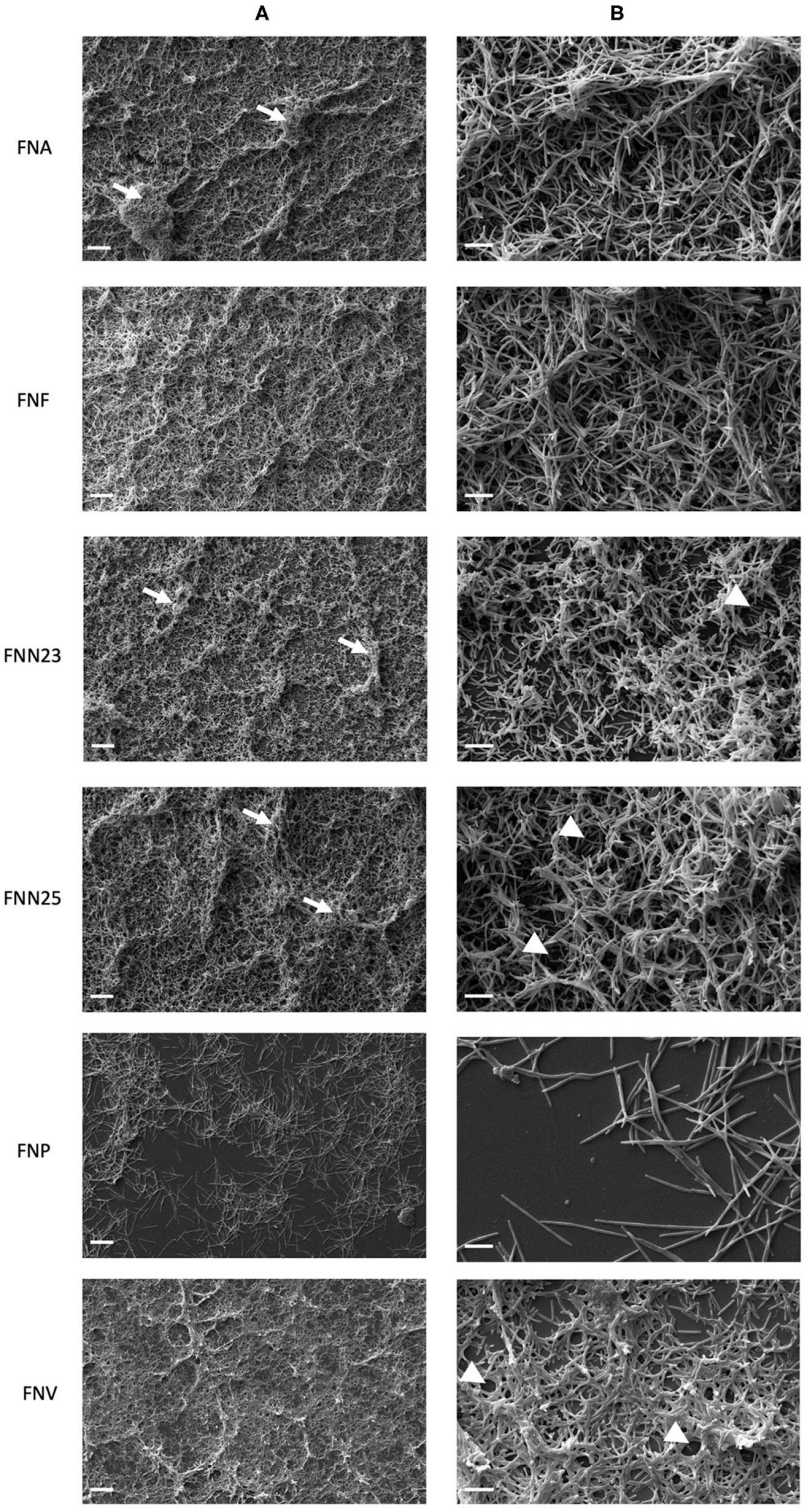
Micrographs of single-subspecies *F. nucleatum* biofilms grown on poly-L-lysine coated Thermanox coverslips. **(A)** Micrographs showing differences in biofilm architecture. White arrows indicate bacterial aggregates within the biofilm. 1000X magnification, scale bar 20 μm. **(B)** Micrographs showing cell-to-cell cohesion. White arrow heads indicate presumed water channels. 5000X magnification, scale bar 5 μm. Biofilms from two independent experiments grown in duplicates were imaged and representative micrographs are shown.

Thermanox coverslips coated with poly-L-lysine were chosen as the surface for biofilm analysis by SEM based on CLSM results, which showed higher biofilm thickness on this type of surface. Overall, high magnification (1000X, Figure 3A) revealed uneven layers of biofilm with raised areas and water channels. FNA, FNF, and FNN25 formed continuous, multi-layered biofilms with visible aggregates, whilst FNN23 formed thinner biofilms, mostly observed as mono-layers with smaller aggregates. Biofilms formed by FNV appeared as flat, continuous mono-layers. Again, FNP did not form a continuous layer of biofilm, but individual bacteria and small pre-biofilm aggregates were observed.

Cell-to-cell cohesion was observed in more detail under 5000X magnification (Figure 3B). In all biofilms, bacterial cells were found to cohere with neighbouring cells either in a parallel fashion, or cells were intertwined. Interestingly, all analysed biofilms seemed to be lacking extracellular matrix (ECM). Taken together, biofilm architecture visibly differed among *F. nucleatum* subspecies with regard to thickness and formation of aggregates and water channels. Cell-to-cell cohesion did not seem to differ among subspecies.

### Conservation of Adhesion Protein Orthologues Varies Among *F. nucleatum* Subspecies

To investigate possible differences in adhesion proteins amongst the subspecies, which might explain the observed lack of biofilm formation in FNP, analysis of the bacterial genomes was carried out. Sequence alignments of Aid1, CmpA, FadA, Fap2, FomA, RadD, and YadA from FNN25 identified orthologous proteins in the *F. nucleatum* ATCC subspecies genomes publicly available on EnsemblBacteria (Release 52) [29]. As expected, FNN23 orthologues were highly conserved (identity >90%). In contrast, our approach did not identify any Aid1, FomA, and RadD orthologues in FNV and any FadA and YadA orthologues in FNA. The genome of FNF was not available on this database.

**Figure 4:**
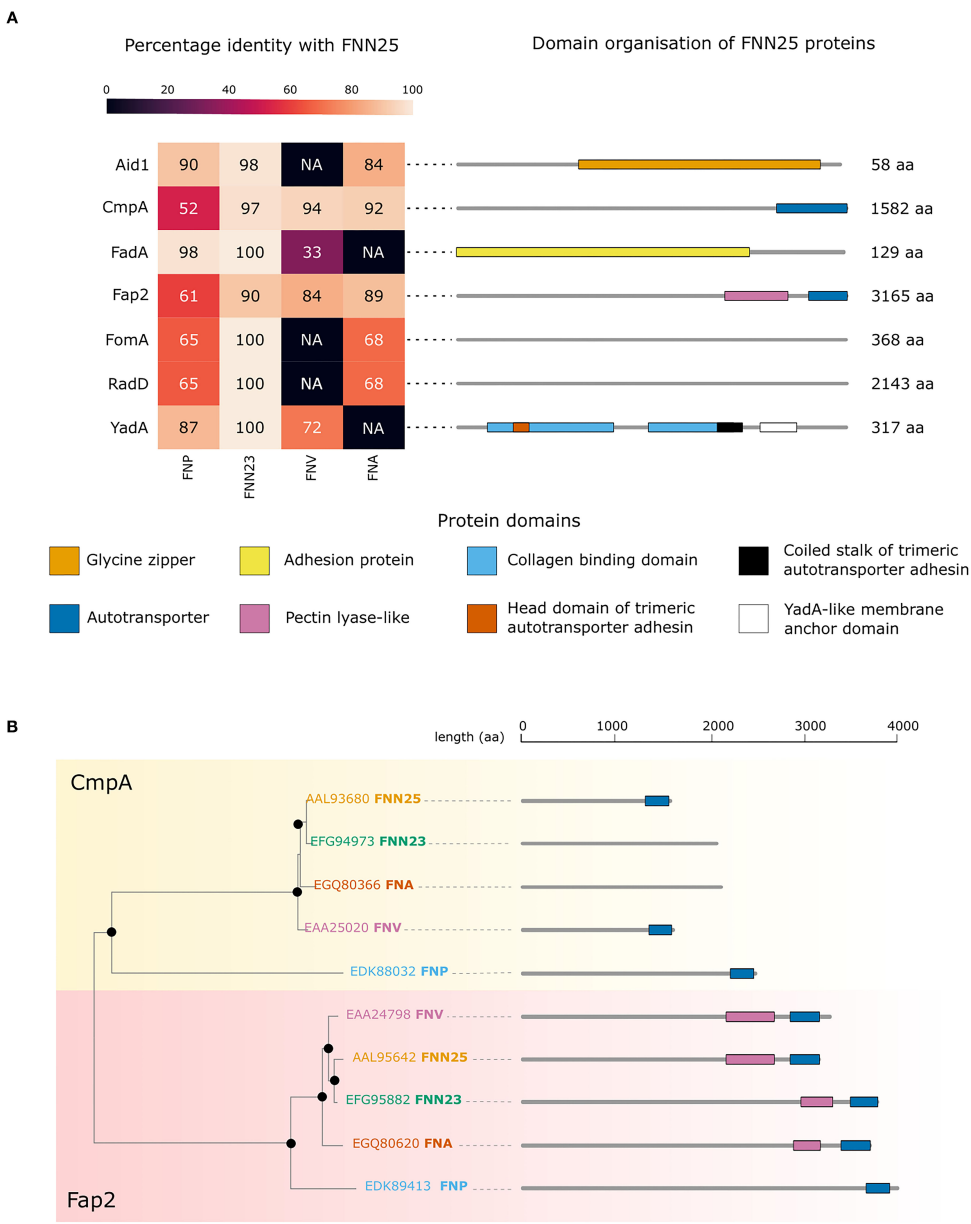
Bioinformatic analysis of adhesion proteins in *F. nucleatum* subspecies. **(A)** Conservation of adhesion proteins in ATCC strains of *F. nucleatum*. The heatmap shows the percentage of identity with the FNN25 adhesion proteins Aid1, CmpA, FadA, Fap2, FomA, RadD, YadA. Protein length and domain organisation are indicated on the right for each of FNN25 proteins. The domains are indicated and coloured differently: glycine zipper (orange); autotransporter (dark blue); adhesion protein (yellow); pectin lyase-like (pink); collagen binding domain (light blue); head domain of trimeric autotransporter adhesin (dark orange); coiled stalk of trimeric autotransporter adhesin (black); YadA-like membrane anchor domain (white). **(B)** CmpA and Fap2 phylogenetic tree. The CmpA branch is highlighted in yellow, the Fap2 branch is highlighted in pink. Black circles represent bootstrap values > 95. FNN25 proteins are coloured in orange; FNN23 proteins in green; FNA proteins in dark orange; FNV in pink; FNP in light blue. Protein structures and lengths are outlined on the right of the tree. Autotransporter domains are coloured in blue, pectin lyase-like domains in pink. Figures were drawn with seaborn (version 0.11.2) and R (version 4.1.2).

Orthologues of all the considered proteins were found in FNP. Interestingly, CmpA and Fap2 orthologues were identified in all subspecies; however, FNP orthologues were less conserved with the lowest identity percentage among the four analysed subspecies (Figure 4A). Annotation of the protein domains highlighted variability in protein domains and length of proteins. Interestingly, both CmpA and Fap2 presented an Autotransporter domain in FNN25 (Figure 4A). Phylogenetic analysis of CmpA and Fap2 shows that the respective orthologues in all the subspecies are highly conserved, except for the FNP proteins, which are more distantly related (Figure 4B).

## Discussion

*F. nucleatum* as a commensal microorganism, opportunistic pathogen, and emerging oncobacterium in the oral cavity and at extraoral sites has received considerable attention in recent years [16]. Individual subspecies have been differentially related to oral health and disease: FNN and FNA have been associated with disease [34–36]; while FNF and FNV have been identified in healthy sites [13, 34]; interestingly, FNP is found in both healthy [34] and diseased periodontal tissues [35].

Differential involvement of subspecies in health and disease might suggest differences in their virulence properties. It is increasingly evident from *in vitro* studies that differences in pathogenicity among *F. nucleatum* subspecies exist, such as differences in coaggregation with other oral bacteria and biofilm formation in multispecies biofilm models [19]. To the authors' knowledge, formation of single-subspecies biofilms by *F. nucleatum* ATCC type strains has not been addressed in the literature to date, and data presented in this study showed for the first time that not all *F. nucleatum* subspecies have the ability to form stable single-subspecies biofilms. In our study, FNP did not form biofilms but only small bacterial aggregates. In support of this finding, similar results were obtained by Karched et al., who showed poor single-subspecies biofilm formation by FNP, whilst a strong autoaggregating ability was shown in this subspecies [37].

Biofilm stability of *F. nucleatum* subspecies has not been addressed in the literature to date. The present study showed considerable biomass variation likely caused by unstable biofilm formation. Under physiological conditions in the oral cavity, *F. nucleatum* grows as a component of complex multi-species biofilms in the presence of multiple binding partners, especially early colonisers [38]. Periasamy et al. showed that subspecies *polymorphum* formed stable biofilms with the early coloniser *Actinomyces naeslundii*, however, it did not form single-subspecies biofilms in saliva [39]. This further supports our observation that FNP does not form stable biofilms on its own. Another factor, which was not considered in the present study, is pH: its manipulation and increase to 8.2 was found to induce biofilm growth in subspecies *polymorphum* [40, 41]. Investigating the impact of pH on biofilm formation by all subspecies may be an important parameter to include in future experiments.

In our study, formation of stable biofilms was also substrate-specific. First, surfaces were coated with AS, which is used to mimic *in vivo* conditions and improve adhesion of bacteria to substrates [23]. The advantage of using AS is the elimination of potentially confounding effects of human salivary components and their natural fluctuations, such as antimicrobial peptides, presence of other bacterial species and their products, and extrinsic or systemic factors [42]. AS significantly supported biofilm formation only in case of FNN25 grown on glass and plastic and FNV grown on plastic surfaces. To the best of the authors' knowledge, *F. nucleatum* single-species biofilm formation on AS coated surfaces has not been reported in the literature to date, however Tavares et al. showed stable single-species biofilm formation by FNF on plastic surfaces coated with human saliva [43]. Using human saliva in future studies of *F. nucleatum* single-species can help to understand whether and how specific salivary properties may influence the selection and growth of certain subspecies in the oral cavity.

Secondly, surfaces coated with fibronectin, a glycoprotein present in physiological fluids such as plasma and saliva [44], were covered with an intermediate amount of biofilm biomass by all subspecies but FNP. In a previous study, *F. nucleatum* was shown to adhere to fibronectin [45] and more specifically, subspecies *polymorphum* ATCC 10953 was found to adhere to fibronectin-coated gingival epithelial cells and fibronectin-coated coverslips as single cells, whilst biofilm formation was not investigated. This supports our finding that single FNP cells adhere to fibronectin coated surfaces, however, studies analysing FNP biofilm formation on fibronectin coated surfaces are missing.

Thirdly, gelatin, a derivative of collagen found in the extracellular matrix of tissues, is a commonly used polymer for coating tissue culture vessels [26]. Benn et al. reported that adhesion of *Escherichia coli* (*E. coli*) was improved by gelatin, but was dependent on specific buffering conditions and bacterial strains [46]. In our study, however, gelatin supported only a low amount of biofilm formation and may therefore not be a recommendable coating agent for *F. nucleatum* biofilms. Additionally, surfaces coated with poly-L-lysine, a cationic coating agent promoting bacterial adhesion by electrostatic interactions as mostly shown in *E. coli* studies [46, 47], led to higher amounts of biofilm in the present study seen microscopically (Figures 2, 3), but this was not statistically significant in CV assays (Figure 1). Nevertheless, we recommend poly-L-lysine as a biofilm-supporting coating agent for *F. nucleatum*.

Apart from surface coatings, surface roughness was also found to promote biofilm formation on multiple types of surfaces by providing a larger surface area for adhesion of bacteria and also protecting adherent bacteria from detachment by shear forces [48]. In this study, the highest mean amount of biomass on glass surfaces was measured on sandblasted glass coverslips. Sandblasting, however, makes glass opaque, rendering biofilm analysis by conventional CLSM challenging if an inverted CLSM is not available. Another material frequently used for oral biofilm assays is hydroxyapatite (HA) [49], mimicking dental enamel surfaces. *In vivo*, periodontal biofilms predominantly adhere to enamel, dentine [50] and cementum [51]. In future studies, utilising dental tissue substrates such as dentine slices or other relevant surfaces such as titanium used in dental implant manufacturing, in combination with inverted CLSM [52] or atomic force microscopy [53, 54] could provide more detailed information on bacterial adhesion and biofilm architecture.

Assessment of the architecture of single-subspecies biofilms showed observable differences among subspecies. Similar observations were made in a previous study investigating *Staphylococcus aureus* and *Pseudomonas aeruginosa*, showing strain-specific differences such as large mushroom-like structures, aggregates and differences in thickness [55]. Possible underlying reasons for differences in architecture might be differences in utilisation of available nutrients by subspecies affecting biofilm maturation or subspecies-specific combination of outer membrane proteins influencing coaggregation.

Of note, biofilm structure also differed between microscopy techniques in our study. For example, FNA biofilms analysed with CLSM appeared as mono-layered structures, while FNA biofilms imaged with SEM were shown to be multi-layered. This may have been due to the fixation procedures affecting the degree of biofilm detachment. Fixation preserves cell morphology and the native structure of biofilms [56], however the degree of paraformaldehyde fixation might have been insufficient in our study due to a short fixation period. Longer incubation in paraformaldehyde [52, 56] and comparison with other types of fixatives in future studies may resolve this issue.

In general, biofilm sample preparation for imaging is often a destructive process, involving multiple washing steps, which may affect biofilm structure and introduce artefacts [57]. Presumed water channels, which were observed in the studied biofilms, could also be considered as such artefacts. Additionally, ECM was not observed in any of the samples assessed in this study. Lack of ECM is likely attributed to the preparation technique for microscopy, as it was demonstrated that biofilm fixation and dehydration for SEM can damage ECM [58]. Presence of ECM in *F. nucleatum* biofilms has been observed in a number of studies, suggesting that *F. nucleatum* forms biofilms according to the definition by Costerton et al. [59]. Ali Mohammed et al. characterised overall composition of ECM in single-species biofilms formed by *F. nucleatum* ssp. *nucleatum* (ATCC 25586) [60]. Subsequently, ECM proteins of the same subspecies were further analysed [61]. Tavares et al. reported ECM surrounding *F. nucleatum* ssp. *fusiforme* cells in single-species biofilms [43]. However, the ECM of the remaining *F. nucleatum* subspecies has not been studied in a single-species biofilm context.

In order to address the limited ability of FNP to form stable biofilms, bioinformatic analysis of known adhesion proteins was performed and showed that a number of orthologues found in this subspecies have very low identity when compared to FNN25, a biofilm forming strain often used as a control in biofilm models [19]. The analysed adhesion proteins RadD, Aid1 [20, 62], Fap2 [63], FadA [21], and CmpA [64] were mainly reported to adhere to other oral pathogens, however they have not been studied in relation to autoaggregation and biofilm formation within the same *F. nucleatum* subspecies. FomA binds to human protein statherin found in saliva [65]. The YadA-like protein, which was originally reported as an adhesin and virulence factor in *Yersinia* species and was identified in *F. nucleatum* [66], adheres to fibronectin and collagen present in the salivary pellicle and extracellular matrix of human cells [22].

Based on our analysis, all proteins were found in the FNP genome, however four of these (CmpA, Fap2, FomA, and RadD) were not well-conserved. Only two proteins, CmpA and Fap2 were detected in all subspecies and were found to share a common autotransporter domain in FNN25. In addition, our *in silico* analysis found the autotransporter domain in Fap2 in all subspecies. Hence, one may speculate that these adhesion proteins and perhaps the autotransporter domain are important for cell-cell adhesion in single-subspecies biofilms. CmpA and Fap2 had a very low identity in FNP, and this subspecies was the most distant in the phylogenetic tree. This might indicate an inability to form stable single-subspecies biofilms. It is, however, important to note that these bioinformatic results have to be considered with caution and no final conclusions can be drawn due to the small scale of the analysis. Only a selected set of adhesion proteins reported in the literature was analysed and it is likely that other putative adhesion proteins involved in biofilm formation remain to be discovered.

With the method employed here, no autotransporter domains for CmpA were detected in subspecies FNA and FNN23. Additionally, despite a high identity of the YadA-like protein in FNP, a general lack of enhanced adhesion of FNP to fibronectin and gelatin in our study might suggest that YadA in FNP does not play a key role in adhesion to these proteins. Mutagenesis studies could be performed in the genetically modifiable strain FNN23 in order to validate these findings. Mutants lacking each of the above-mentioned adhesion proteins could be studied regarding their adhesion, autoaggregation and biofilm forming ability.

Many authors have utilised planktonic cultures in pathogenicity studies of *F. nucleatum*, however this might not reflect true virulence of this anaerobic microorganism, as virulence genes have been shown to be upregulated in biofilms [67, 68]. Availability of *F. nucleatum* single-subspecies biofilm characterisation data might help researchers to perform more biologically relevant *F. nucleatum* pathogenicity studies using single-subspecies biofilms. Our experiments showed that in general both glass and plastic surfaces supported biofilm growth and thus can be utilised for studies of *F. nucleatum* subspecies biofilms when transparent substrates are required. Additionally, surface coatings do not seem to be necessary when quantity of the biofilm is not a priority.

Whilst we appreciate that behaviour of laboratory type strains might differ significantly from clinical isolates obtained from patients, we believe that it is important to first characterise virulence properties of subspecies of *F. nucleatum*, represented by widely available ATCC type strains, to build a knowledge base, which can later be utilised to study virulence of specific clinical isolates. As biofilm formation is one of the virulence properties of bacteria, understanding the differences in pathogenicity of individual *F. nucleatum* subspecies may reveal new virulence factors as novel therapeutic targets for prevention and treatment of *F. nucleatum*-mediated infections and diseases.

## Data Availability Statement

The original contributions presented in the study are included in the article/Supplementary Material, further inquiries can be directed to the corresponding author/s.

## Author Contributions

MM carried out experiments and data analysis and wrote the manuscript. DB performed bioinformatic analysis and wrote the corresponding parts of the manuscript. MG, SK, IC, and JH were involved in planning experiments, supervised the project, and contributed to the interpretation of the results. JH provided support with statistical analysis and figure preparation. All authors provided critical feedback on the manuscript. All authors contributed to the article and approved the submitted version.

## Funding

This study was funded by a grant provided by Birmingham Community Healthcare NHS Foundation Trust. Publication of this study was possible thanks to funds provided by the University of Birmingham, Birmingham, UK.

## Conflict of Interest

The authors declare that the research was conducted in the absence of any commercial or financial relationships that could be construed as a potential conflict of interest.

## Publisher's Note

All claims expressed in this article are solely those of the authors and do not necessarily represent those of their affiliated organizations, or those of the publisher, the editors and the reviewers. Any product that may be evaluated in this article, or claim that may be made by its manufacturer, is not guaranteed or endorsed by the publisher.
