# Supplementary material for: Fusobacterium nucleatum Subspecies Differ in Biofilm Forming Ability in vitro
Source: Front Oral Health. 2022 Mar 15;3:853618. doi: 10.3389/froh.2022.853618 (PMC8967363; doi:10.3389/froh.2022.853618)
Supplement: Supplementary file 1 [file Image_1.pdf]

Supplementary Material

Supplementary figure 1: “Representative 2D CLSM images of *F. nucleatum* biofilms” can be found on the next page.

Bioinformatic supplementary material can be found in an enclosed zipped folder (SupplementaryMaterials_Bioinformatics).

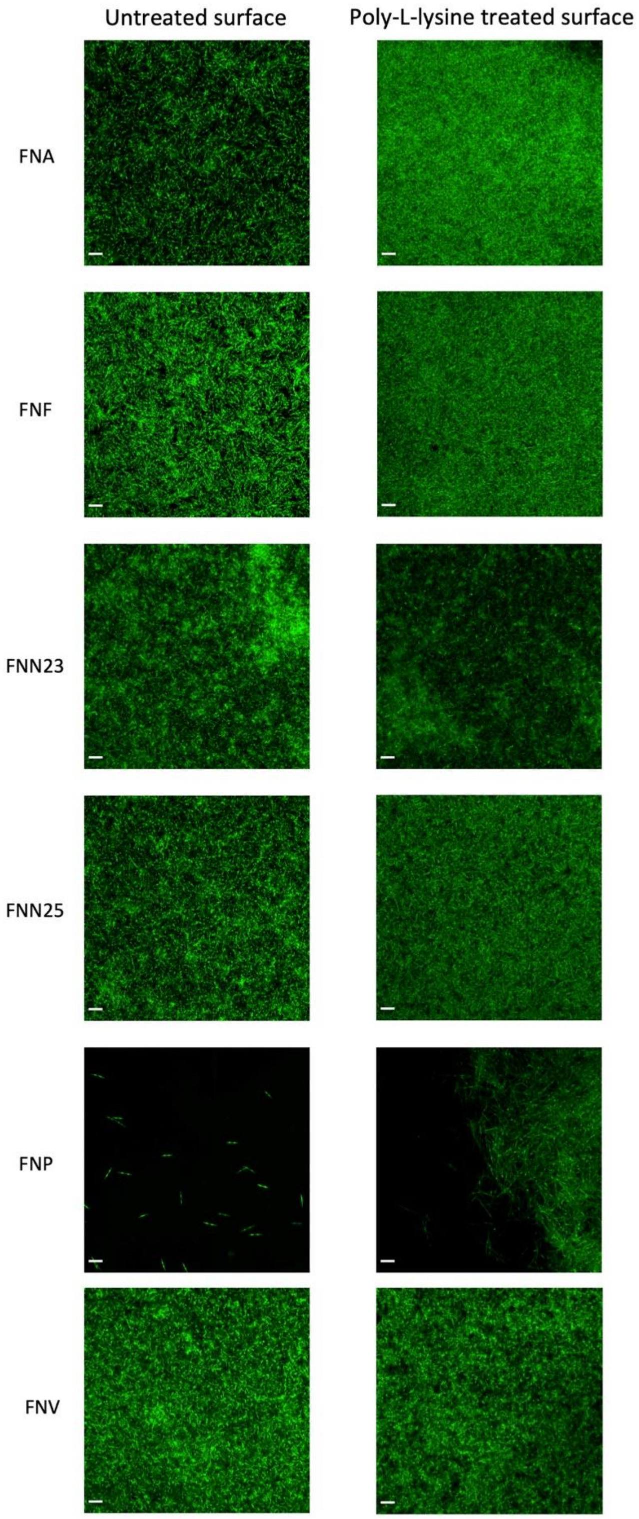

Supplementary Figure 1: Representative 2D images of single-subspecies *F. nucleatum* biofilms imaged by CLSM. Biofilms were grown on untreated and poly-L-lysine coated plastic surfaces. Note the absence of a continuous biofilm layer in FNP: only single cells were observed on the untreated surface while a bacterial aggregate was observed on the poly-L-lysine treated surface. Scale bar: 10 µm.
